# Dynamic Evolution of Bacterial Ligand Recognition by Formyl Peptide Receptors

**DOI:** 10.1093/gbe/evad175

**Published:** 2023-09-30

**Authors:** Nicole M Paterson, Hussein Al-Zubieri, Joseph Ragona, Kristin M Kohler, Juan Tirado, Brian V Geisbrecht, Matthew F Barber

**Affiliations:** Institute of Ecology and Evolution, University of Oregon, Eugene, Oregon, USA; Centers for Disease Control and Prevention, Atlanta, Georgia, USA; Institute of Ecology and Evolution, University of Oregon, Eugene, Oregon, USA; Department of Biochemistry and Molecular Biophysics, Kansas State University, Manhattan, Kansas, USA; Institute of Ecology and Evolution, University of Oregon, Eugene, Oregon, USA; Institute of Ecology and Evolution, University of Oregon, Eugene, Oregon, USA; Department of Biochemistry and Molecular Biophysics, Kansas State University, Manhattan, Kansas, USA; Institute of Ecology and Evolution, University of Oregon, Eugene, Oregon, USA; Department of Biology, University of Oregon, Eugene, Oregon, USA

**Keywords:** molecular evolution, positive selection, immunity, receptors, bacteria, pathogen

## Abstract

The detection of invasive pathogens is critical for host immune defense. Cell surface receptors play a key role in the recognition of diverse microbe-associated molecules, triggering leukocyte recruitment, phagocytosis, release of antimicrobial compounds, and cytokine production. The intense evolutionary forces acting on innate immune receptor genes have contributed to their rapid diversification across plants and animals. However, the functional consequences of immune receptor divergence are often unclear. Formyl peptide receptors (FPRs) comprise a family of animal G protein–coupled receptors which are activated in response to a variety of ligands including formylated bacterial peptides, pathogen virulence factors, and host-derived antimicrobial peptides. FPR activation in turn promotes inflammatory signaling and leukocyte migration to sites of infection. Here we investigate patterns of gene loss, diversification, and ligand recognition among FPRs in primates and carnivores. We find that FPR1, which plays a critical role in innate immune defense in humans, has been lost in New World primates. Amino acid variation in FPR1 and FPR2 among primates and carnivores is consistent with a history of repeated positive selection acting on extracellular domains involved in ligand recognition. To assess the consequences of FPR divergence on bacterial ligand interactions, we measured binding between primate FPRs and the FPR agonist *Staphylococcus aureus* enterotoxin B, as well as *S. aureus* FLIPr-like, an FPR inhibitor. We found that few rapidly evolving sites in primate FPRs are sufficient to modulate recognition of bacterial proteins, demonstrating how natural selection may serve to tune FPR activation in response to diverse microbial ligands.

**Significance**

Host immune receptors may undergo natural selection in response to antagonism by pathogens, yet the consequences of these mutations are often unclear. We find that mammalian formyl peptide receptors have experienced rapid diversification and gene loss which modulates binding to both activating and inhibitory pathogen ligands. These results demonstrate how selection can have pleiotropic effects on pathogen detection and immune activation during receptor evolution.

## Introduction

Formyl peptide receptors (FPRs) encompass a family of vertebrate G protein–coupled receptors (GPCRs) that play crucial roles in the recruitment and activation of leukocytes during infection (Kim et al. 2009; Kretschmer et al. 2010; Bloes et al. 2015; Bufe et al. 2015; Weiß and Kretschmer 2018; Leslie et al. 2020). FPRs were originally identified when researchers observed leukocyte migration toward *N*-formylated peptides, which are present in bacterial and mitochondrial proteins but not eukaryotic nuclear-encoded proteins (Schiffmann et al. 1975). This led to the discovery of FPR1 as a host receptor which detects formylated peptides (Pike et al. 1980; Zigmond 1981). Since then, additional microbial and host-derived ligands have been identified for specific FPR homologs. Of the three FPRs in humans, each has been shown to possess a unique ligand-binding profile with a range of downstream responses (Karlsson et al. 2009; Kretschmer et al. 2010; Schepetkin et al. 2014). For example, recognition of lipoxin A by FPR2 leads to the suppression of inflammatory signaling, whereas binding of bacterial-specific formylated peptides by FPR1 results in induction of the inflammatory response and cell chemotaxis toward ligand source (Le et al. 2002; John et al. 2007; Schepetkin et al. 2014).

Upon FPR activation in neutrophils, these crucial innate immune cells contribute to pathogen clearance through phagocytosis, release of toxic granule molecules, and production of reactive oxygen and nitrogen species (Önnheim et al. 2014; Bufe et al. 2015). Neutrophils constitute roughly 50% of circulating bloodstream leukocytes and are capable of detecting nanomolar concentrations of pathogen-derived peptides via FPR1 and FPR2 (Le et al. 2002; Fu et al. 2006). Natural killer cells, monocytes, and macrophages also express high levels of FPRs which contribute to activation and chemotaxis of these immune cell types (Crouser et al. 2009; Kim et al. 2009; Leslie et al. 2020).

Immune receptors are under persistent selective pressure to detect an array of rapidly evolving microbial ligands (Daugherty and Malik 2012; Aleru and Barber 2020). Beneficial mutations that enhance immune responses are expected to spread rapidly in host populations via positive selection. Previous studies have detected signatures of positive selection in FPR1 and FPR2 in the mammalian lineage through observation of elevated rates of nonsynonymous to synonymous substitutions (dN/dS) at several codon sites (Muto et al. 2015). Many other proteins involved in host defense against pathogens including Toll-like receptors (TLRs), major histocompatibility complex (MHC) genes, and transferrin family genes have been subject to repeated positive or balancing selection during mammalian evolution (Hughes and Nei 1989; Sawyer et al. 2005; Barreiro et al. 2009; Barber and Elde 2014; Barber et al. 2016; Levin and Malik 2017). FPRs play a central role in innate immunity, and heightened molecular signatures of positive selection suggest sequence variation may play an important functional role in immunological adaptation in mammals. In the present study, we integrate genetic and experimental approaches to investigate the consequences of FPR variation between primate and carnivore species. Our findings indicate that rapid evolution of FPR orthologs between closely related species can have major impacts on recognition of distinct pathogen ligands.

## Results

### Loss of FPR1 in New World Primates

**Fig. 1.**
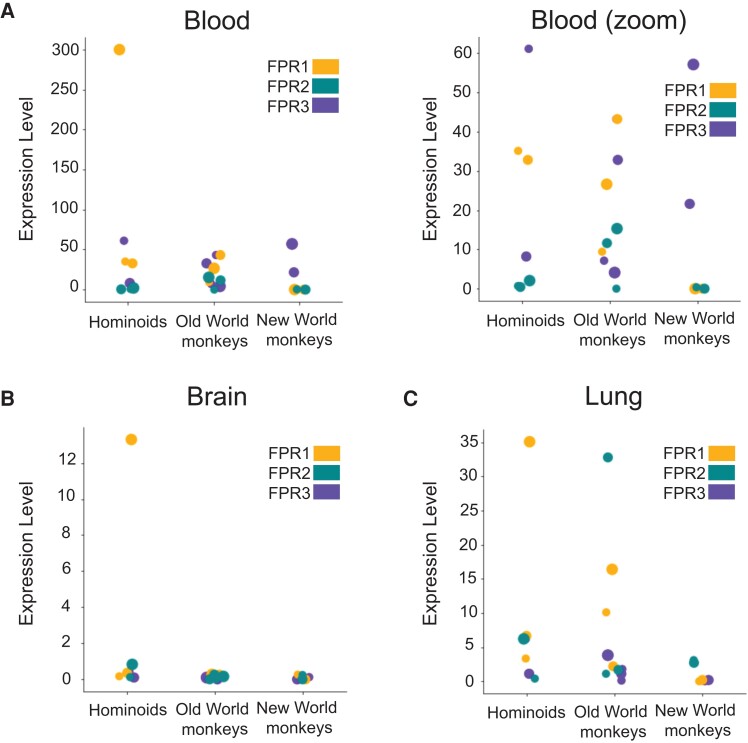
Expression of FPR paralogs among simian primates. FPR expression levels in (*A*) whole blood, (*B*) brain, and (*C*) lungs across hominoid (human and chimpanzee), Old World monkey (pig-tailed macaque, crab-eating macaque, baboon, and mangabey), and New World monkey (marmoset, squirrel monkey, and owl monkey) species. Data obtained from the AceView NIHTPR RNA-Seq data set.

**Fig. 2.**
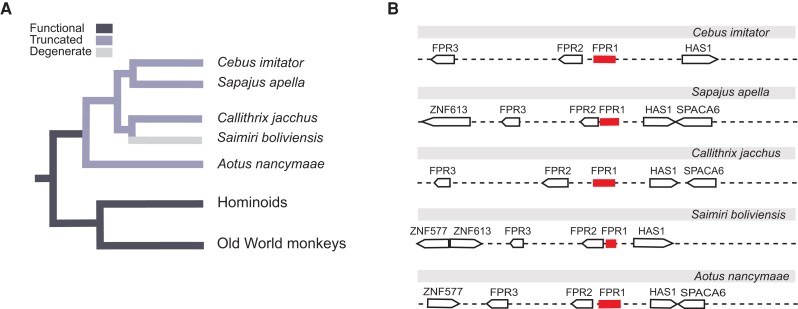
Loss of *FPR1* in New World primates. (*A*) Evidence for inactivating mutations in *FPR1* within the New World monkey lineage. *Saimiri boliviensis* displayed the most significant degeneration in this region, encoding only a short 271 nucleotide sequence with significant *FPR1* similarity and no apparent start or stop codons. (*B*) Conserved synteny of the FPR gene cluster across New World monkeys.

To begin to investigate the consequences of FPR evolution on immune functions, we compiled a data set of FPR homologs from various primate and carnivore species. Unexpectedly, we found evidence for a loss of FPR1 expression in New World primates demonstrated by a lack of FPR1 cDNA detectable in whole blood, brain, lung, and other RNA-Seq data from AceView (Thierry-Mieg and Thierry-Mieg 2006) (fig. 1). This lack of expression suggested that the *FPR1* gene may have been downregulated, lost, or pseudogenized in New World monkeys. Further investigation identified pseudogenes as well as a lack of annotated or homologous *FPR1* New World monkey genes in the NCBI database. These incomplete gene sequences nonetheless shared sequence identity with related *FPR1* genes (fig. 2). To determine whether *FPR1* genes in New World monkeys were subject to gene degradation or unexpressed or degraded mRNA, we scanned available New World monkey genomes using a BLAT search for regions of similarity to *FPR1* genes both manually and by use of the bioinformatics tool GAMMA (Stanton et al. 2021). We tested for the presence of predicted exons using the GENESCAN tool (Burge and Karlin 1997) but failed to identify exons for any of the gene regions in New World monkeys containing significant similarity to marmoset *FPR1*. We aligned regions identified as putative pseudogenes in New World monkeys to the human *FPR1* reference and the marmoset *FPR1* pseudogene sequences (which are available as high coverage, well-annotated entries in the NCBI database). *Sapajus apella*, *Cebus imitator*, *Saimiri boliviensis*, and *Aotus nancymaae* have substantial similarity to FPR1 at syntenic loci in their genomes (adjacent to the *FPR2* and *FPR3* genes on chromosome 19) but lack one or more features of a functional gene (fig. 2*A* and *B*). The *S. boliviensis* pseudogene is the most striking, as this region has the least conservation (77.5% identity to marmoset *FPR1* in 271 nucleotide region located on the plus-strand adjacent to the *FPR2* and *FPR3* genes on chromosome 19), lacks evidence of a start or stop codon, and lacks significant sequence identity to marmoset *FPR1* outside of the 271 nucleotide region. It should be noted that each of these genomes possesses different levels of coverage and/or one or more builds. However, *S. boliviensis*, *A. nancymaae*, and *Callithrix jacchus* have multiple builds with high coverage (*S. boliviensis*: 2 builds, most recent 111× coverage; *A. nancymaae*: 4 builds, most recent 132×; *C. jacchus*: 11 builds, most recent 40× coverage). We probed the genomes listed against gelada *FPR1* nucleotide sequence obtained from NCBI with GAMMA, using the identity threshold of 50%, and obtained no hits for any of the primate genomes tested. Collectively, these findings indicate that the *FPR1* immune receptor has been lost in New World primates.

### Rapid Divergence of FPR Ligand-Binding Domains in Primates and Carnivores

**Fig. 3.**
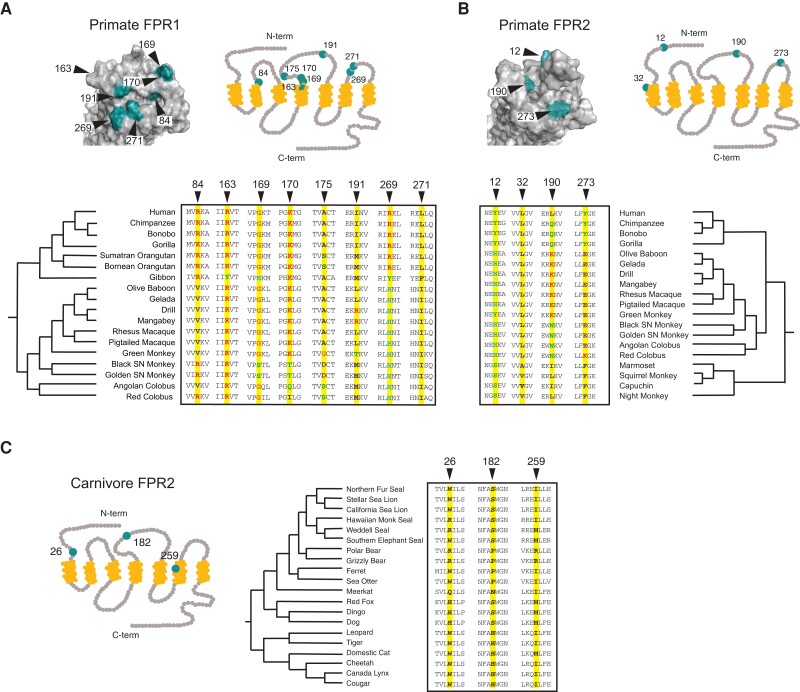
Evidence of repeated positive selection acting on extracellular domains of primate and carnivore FPRs. Sites in (*A*) primate FPR1, (*B*) primate FPR2, and (*C*) carnivore FPR2 with elevated dN/dS as determined by PAML and HyPhy. Residues of the transmembrane domain are denoted in yellow on the protein diagram, with the majority of high dN/dS sites (teal) located in the extracellular (top) ligand-binding loops. Species used for phylogenetic analyses and are indicated in the phylogenies for each gene. Sites in with elevated dN/dS as determined by PAML and HyPhy are denoted with black arrows in the amino acid alignment.

To test whether sequence divergence in FPRs may be driven by natural selection, we performed phylogenetic analysis by maximum likelihood (PAML) on publicly available primate and carnivore sequences. Primate and carnivore FPR trimmed gene sets were analyzed using codeml from the PAML package (Yang 2007), and omega values were assessed for statistical significance by chi-squared analysis. Our reasoning for inclusion of carnivores in addition to primates was due to Carnivora encoding only a single FPR homolog, FPR2. We were curious whether reduced gene copy number in this clade could alter the strength or patterns of natural selection compared with other taxa. Our analysis revealed evidence for positive selection acting on FPR1 and FPR2 genes in primates, largely consistent with previous studies across mammals, particularly in the extracellular loops which participate in ligand binding (Muto et al. 2015) (fig. 3). Notably, positions 170, 191, and 271 in FPR1 were also identified in this previous study. We performed additional testing for branch-site episodic positive selection using aBSREL (Smith et al. 2015) and found evidence of heightened selection on the branch of the phylogenetic tree leading to the New World monkey FPR2. Phylogenetic analysis of positive selection at the codon level in carnivores revealed similar patterns of elevated dN/dS in FPR2. The sites under positive selection that appeared in carnivores mapped to the N-terminus and the third and fourth extracellular domains, the exact regions that appear to be undergoing selection in primates as well (fig. 3). To determine how these observed evolutionary changes may influence FPR immune functions, we next took an empirical approach to assess interactions between FPR homologs and known microbial ligands.

### Recognition of Bacterial Ligands by Mammalian FPRs

To assess the functional consequences of sequence variation in primate FPRs, we focused on interactions with the pathogenic bacterium *Staphylococcus aureus* due to its expression of both inhibitors and activators of FPRs (Prat et al. 2009; Kretschmer et al. 2010; Stemerding et al. 2013; Koymans et al. 2017). *S. aureus* is a Gram-positive bacterium known for its multiplicity of virulence factors, antibiotic resistance, and ability to infect a broad range of mammals including primates and livestock (Thomer et al. 2016; Balasubramanian et al. 2017; Haag et al. 2019; Kwiecinski and Horswill 2020; Cheung et al. 2021). This adaptable microbe colonizes the nares of roughly 30% of humans and is also a major cause of skin and soft tissue infections, bacterial sepsis, pneumonia, and other life-threatening infections (Thomer et al. 2016; Kwiecinski and Horswill 2020; Cheung et al. 2021). *S. aureus* produces many potent toxins believed to contribute to its virulence, including enteroxins, superantigens, proteases, leukocidins, and alpha-hemolysin (Tam and Torres 2019). *S. aureus* evades host immune responses in part through release of protein inhibitors of TLRs, FPRs, and complement receptors (Postma et al. 2004; Prat et al. 2009; Thammavongsa et al. 2013). Strains sampled from wild gorillas, chimpanzees, green monkeys, and colobus monkeys contain the gene for the virulence factor staphylococcal enterotoxin B (SEB), shown to potently activate FPRs (Schaumburg et al. 2012) and produce formylated peptides that can induce cell migration in neutrophils. *Staphylococcus aureus* also produces a number of molecular inhibitors that directly bind and inactivate FPRs, such as FLIPr and FLIPr-like (Stemerding et al. 2013).

**Fig. 4.**
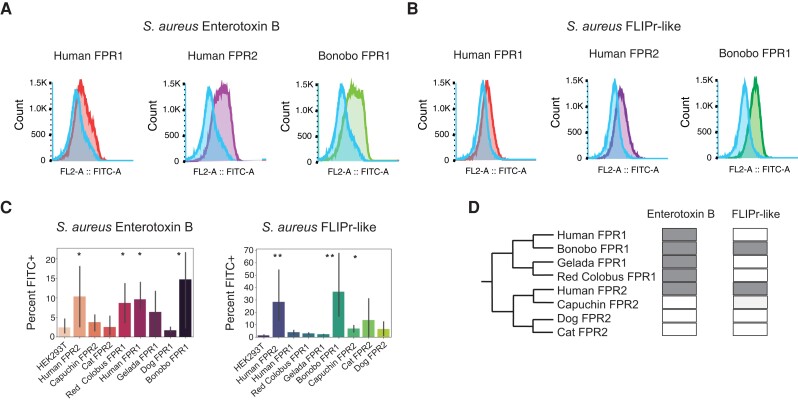
Mammalian FPR variation shapes recognition of activating and inhibitory bacterial ligands. HEK293T cells expressing mammalian FPRs were incubated with (*A*) FITC-conjugated SEB or (*B*) FITC-conjugated *S. aureus* FLIPR-like protein. Samples were then washed and analyzed by flow cytometry. Data from at least three flow cytometry experiments were collected for each species. (*C*) Singlet cells from (*A*) and (*B*) were gated and percent of FL2 (488+) positive cells reported. Error bars represent standard error of the mean based on three experiments. *P < 0.05 and **P < 0.01 compared with HEK293T control. (*D*) Summary of binding results for SEB and FLIPR-like proteins to FPR homologs. Gray indicates a positive detected interaction; white indicates no significant interaction detected.

We tested binding of SEB or FLIPr proteins fluorescently labeled with fluorescein (fig. 4*A* and *B*; supplementary figs. 1 and 2, Supplementary Material online) to human HEK293T cells expressing primate FPRs by flow cytometry. Binding was assessed based on FITC^+^ after incubation and washing of unbound labeled protein. Our results for SEB revealed evidence of binding for many of the receptors tested (fig. 4). All of the primate FPR1 proteins were found to bind SEB, whereas humans were the only species for which FPR2 bound (fig. 4*C*–*F*). The *S. aureus* inhibitor FLIPr-like did not bind FPR proteins with any clear phylogenetic relationship (fig. 4*B*–*D* and *F*). Most strikingly, bonobo FPR1 bound FLIPr-like similarly to human FPR2, not human FPR1, despite sharing 98% sequence identity with human FPR1. We next considered which FPR amino acid positions were most likely to be responsible for binding differences observed. Without crystal structures available for FPRs, we generated homology models using AlphaFold2, which has demonstrated high accuracy that is comparable or better to resolved nuclear magnetic resonance structures at the single Ångström level for protein in solution (Zweckstetter 2021). Our results matched the pattern of binding affinity seen in our experimental results, with strongest binding in FPR2, followed by bonobo and least for human FPR1. Gibbs free energy for ligand docking experiments are often given less weight than the relative values, because the environment where binding occurs plays a strong role in the precise value of the free energy of the binding interaction, so we used Glide docking score as a relative measure of binding affinity to validate the likely accuracy of our predicted structures (Friesner et al. 2004).

The AlphaFold structures were docked to the FPR extracellular region using the seven amino acids that form the region of FLIPr and FLIPr-like N-terminal peptide required for its inhibitory activity using Schrödinger Glide docking software, with the best docking pose in the expected conformation. Because we used a fragment of an intrinsically disordered region of the larger protein, FLIPr-like, we rejected poses where the terminal lysine was not projecting from the pocket and selected the highest binding affinity/lowest Glide score from the docking run. We also ran these same experiments using i-TASSER to predict the structures with the same docking parameters used in Schrödinger Glide. Both experiments produced similar results.

**Fig. 5.**
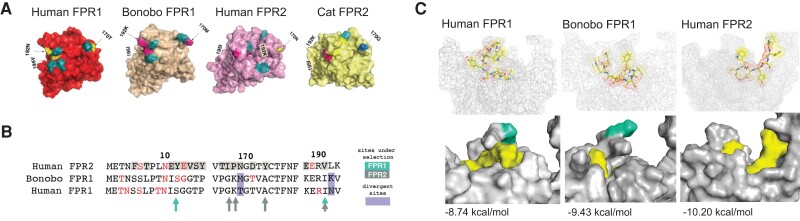
Rapidly evolving surfaces of FPRs contribute to predicted bacterial ligand binding. (*A*) i-TASSER predicted structures for human FPR1, bonobo FPR1, human FPR2, and cat FPR2. Sites exhibiting elevated dN/dS in the extracellular region are indicated in teal. Sites 170 and 190, which are polymorphic in human populations, are also indicated. (*B*) Multiple sequence alignment for sites under selection at FPR extracellular regions of interest. Divergent sites shown in purple highlight differences between human and bonobo FPR1. Sites under selection are indicated by teal (FPR1) and gray (FPR2) arrows. (*C*) Predicted structures of FPR1 reveal an occluding structure that may affect binding of FLIPR-like. Site 170, which is variable between humans and bonobos, is highlighted in teal.

The results of these experiments suggested that FPR1 in humans may have less propensity for FLIPr-like inhibition compared with bonobo FPR1 and human FPR2. Looking closer at the structures revealed a site under selection is likely responsible for this difference in activity: position 170 in FPR1 is a methionine in bonobos, resulting in less obstruction of the binding pocket of FPR1 (fig. 5*A* and *B*). We found the ligand is predicted to bind much deeper in the pocket than FLIPr-like (supplementary fig. 3, Supplementary Material online), suggesting the difference between bonobo FPR1 and human FPR1 activation by f-MLF might not be affected by the occluding structure formed by site 169/170 in the human FPR1 predicted structure (fig. 5*C*).

## Discussion

Our analysis suggests that FPR1 function was lost early in the New World monkey lineage. It is possible this gene loss reflects neutral genetic processes due to functional redundancy among FPR genes. Alternatively, FPR1 loss may have been beneficial under certain conditions due to potential fitness costs associated with FPR1 activity. In this regard, FPR activation has been associated with human disease states such as glioblastoma, breast cancer, and inflammatory diseases (Khau et al. 2011; Cussell et al. 2019; Leslie et al. 2020). At present, the evolutionary processes underlying *FPR1* loss in New World primates remain unclear. Like other immune receptor families (Barber et al. 2017; Águeda-Pinto et al. 2019; Jacquet et al. 2022), gene duplication and loss have occurred periodically during the evolution of the FPR gene family in mammals as illustrated by the wide range of copy number across taxa. Studies in rodents have revealed remarkable functional plasticity in FPRs (Dietschi et al. 2017). This plasticity likely explains in part why these genes have undergone repeated duplication and loss. Future work leveraging animal genetics to explore redundancy and costs of FPR function may aid in resolving these questions.

Our results indicate that bonobo FPR1 binds to *S. aureus* inhibitor FLIPr-like in a manner similar to human FPR2, even though it shares far more sequence similarity with human FPR1. This suggests a small number or even a single amino acid change could be responsible for this difference in activity. On the other hand, mutations that reduce interactions with pathogen inhibitors may also alter binding to crucial FPR agonists. Future studies leveraging site-directed mutagenesis to assess binding to large sets of FPR ligands could improve our understanding of the pleiotropic consequences of receptor variation within and across species. Notably, we observed the presence of several FPR1 polymorphisms in the human population that are predicted to influence bacterial ligand recognition (supplementary fig. 4, Supplementary Material online). The site which forms a "ridge" in bonobo FPR1 relative to humans, T170M, naturally occurs in the human population, as well as an additional T170P polymorphism which occurs at low frequency (supplementary fig. 4, Supplementary Material online). The consequences of these human FPR variants for recognition by FLIPr-like and other bacterial inhibitors remain unknown.

In addition to influencing receptor activation or repression, rapid evolution of FPRs may have additional consequences related to infectious disease susceptibility. It has recently been shown that FPR1 is recognized by the type 3 secretion system of *Yersinia pestis*, mediating leukocyte killing during plague infection (Osei-Owusu et al. 2019). This study further demonstrated that the human *FPR1* R190W polymorphism is protective against *Y. pestis* infection. Thus, future studies exploring the functional consequences of FPR evolution would greatly contribute to our understanding of these crucial immune receptors in health and disease.

## Materials and Methods

### Phylogenetic Analyses

We inferred amino acid sites exhibiting elevated dN/dS using multiple computational methods. Our data set included available nucleotide coding sequences (cDNA) of FPR1 for 18 primate species (human, drill, mangabey, red colobus, black and white colobus, snub-nosed monkey, golden snub-nosed monkey, Sumatran orangutan, Bornean orangutan, gorilla, chimpanzee, bonobo, white-cheeked gibbon, green monkey, crab-eating macaque, pig-tailed macaque, gelada, and olive baboon), with areas of ambiguity and stop codons removed. A gene tree for FPR paralogs was generated with phylogenetics by maximum likelihood (PhyML) with 1,000 bootstraps. Potential sites under positive selection were detected using the PAML package (Yang 2007), which detects signs of positive selection from the frequency of nonsynonymous/synonymous amino acid substitutions at each site (*ω* = dN/dS) based on maximum likelihood. Additional computational methods MEME (Murrell et al. 2012) and FuBar (Murrell et al. 2013) from the Datamonkey adaptive evolution server were cross-referenced, and sites that appeared in more than one analysis with high confidence (*P* < 0.01) were included. aBSREL analysis which tests for branch-site episodic selection was also performed (Smith et al. 2015).

### Cloning and Lentiviral Transduction of FPRs in HEK293T Cells

FPR1 genes for human, bonobo, gelada, and red colobus and FPR2 genes for human, capuchin, dog, and cat were cloned from cDNA (human FPR1 and FPR2), synthesized by Genewiz (gelada and red colobus), or synthesized as gBlocks by IDT (capuchin, dog, and cat) including Kozak sequence and C-terminal Flag-tag. DNA fragments were subsequently cloned into the pBABE lentiviral vector using SLIC or Gibson cloning methods. Expression was verified in cell lines by Western blot using anti-Flag tag antibodies (Monoclonal ANTI-FLAG® M1, Sigma Aldrich #F3040). Surface expression was verified for FPR1 using Thermo Fisher FPR1 polyclonal antibody (PA5-33534), and cell lines with comparable cell surface expression were used for binding experiments. FITC labeling was performed per manufacturer's instructions, and the Thermo Scientific™ Pierce™ Dye Removal Columns (part number 22858) were used to remove excess dye.

### Flow Cytometry

Cells expressing FPRs from primates (as described above) were counted and suspended at 10^5^ cells/ml 4 μg of FITC-labeled FLIPr-like, or SEB proteins were incubated in 100 μl sterile phosphate-buffered saline + 100 nM PMSF at 4 °C with nutation for 1 h, washed 3× with 1 ml ice-cold PBS, and analyzed on a SONY SH800 flow cytometer and assessed for binding by proportion of cells positive for 488+ as compared with negative control (nontransfected HEK cells incubated with fluorescently labeled proteins in parallel with test samples).

### FLIPR-Like Peptide Docking to Human FPRs

Initial structures for analysis were generated using the I-TASSER server, except for human FPR1, bonobo FPR1, and human FPR2, which were also predicted using the CoLab version of AlphaFold2 (Jumper et al. 2021). The AlphaFold2 structures were docked to FFSYEWK (which represents the first seven amino acids of FLIPr and FLIPr-like proteins), whereas the iTASSER structures were docked to FFSYEWK and f-MLF. FFSYEWK was generated in PyMOL (The PyMOL Molecular Graphics System, Version 2.0 Schrödinger, LLC). Peptide docking was run with Schrödinger Glide XP, with sidechain protonation set to represent charged states at pH 7. The Highest ranking docking was used for analysis, with lysine peptides internal to the pocket dismissed (Friesner et al. 2004).

### Detection of FPR1 Pseudogenes in New World Monkeys

New World Monkey FPR1 was queried by BLAST search, followed by command line BLAT search of genomes downloaded from NCBI (*C. jacchus*, *S. boliviensis*, *Cebus capucinus imitator*, *S. apella*, and *A. nancymaae*). Pseudogenes were aligned to closest related extant FPR gene using MUSCLE. Resulting data were analyzed for exons using GENESCAN (Burge and Karlin 1997).

## Supplementary Material

evad175_Supplementary_Data

## Data Availability

Data underlying this article are available in supplementary table 1, Supplementary Material online, and any additional data are available upon request to the corresponding author.
