# Supplementary material for: Dynamic Evolution of Bacterial Ligand Recognition by Formyl Peptide Receptors
Source: Genome Biol Evol. 2023 Sep 30;15(10):evad175. doi: 10.1093/gbe/evad175 (PMC10566242; doi:10.1093/gbe/evad175)
Supplement: evad175_Supplementary_Data [file evad175_supplementary_data.zip › Paterson_FPRs_supplement2023_FINAL.pdf]

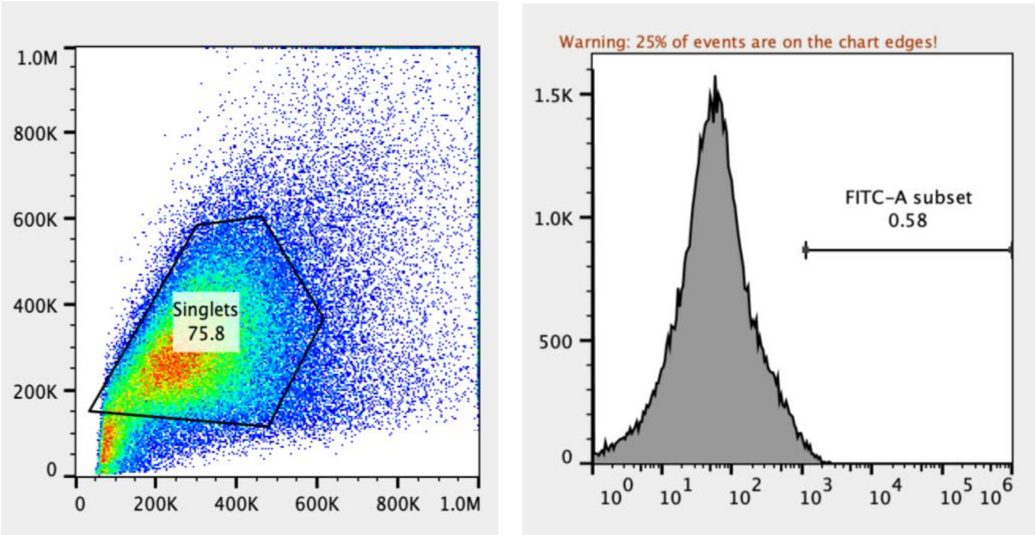

Supplemental Figure 1. Gating strategy for flow cytometry.
Singlets were selected using forwards scatter versus side scatter, of these singlets, (FITC/488)/FL2+ cells were identified using unstained cells as baseline.

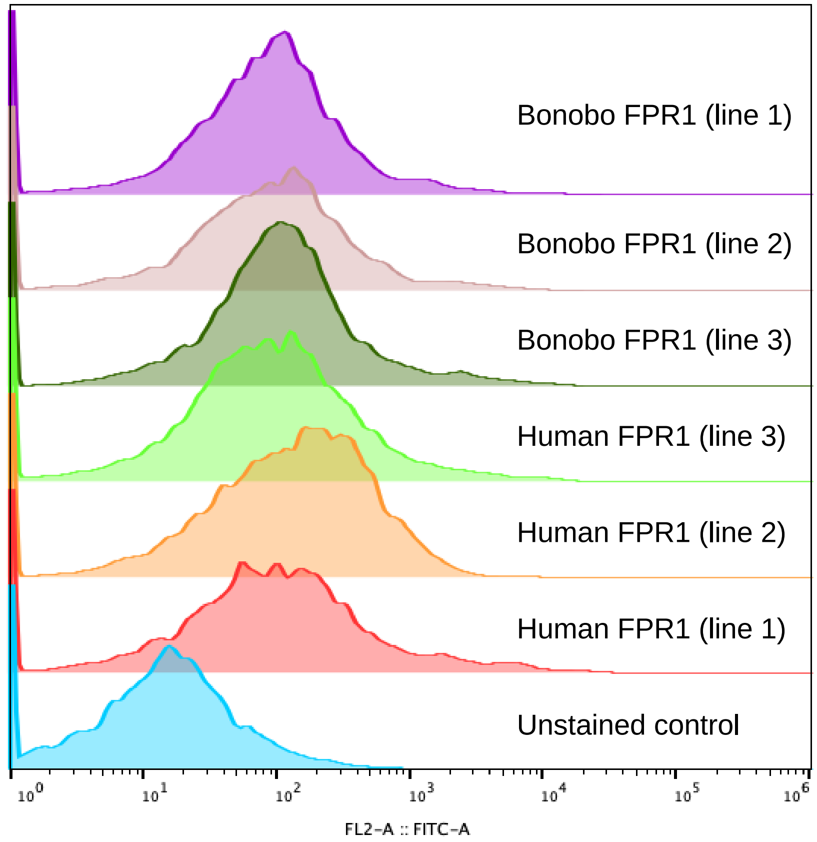

Supplemental Figure 2. Expression of great ape FPR1 in human cell lines.
Human and bonobo FPR1 genes were expressed in human 293T cells by transient transfection and detected using a FITC-conjugated human FPR1 polyclonal antibody via flow cytometry. Three independent cell lines for each species were included, as well as an unstained negative control.

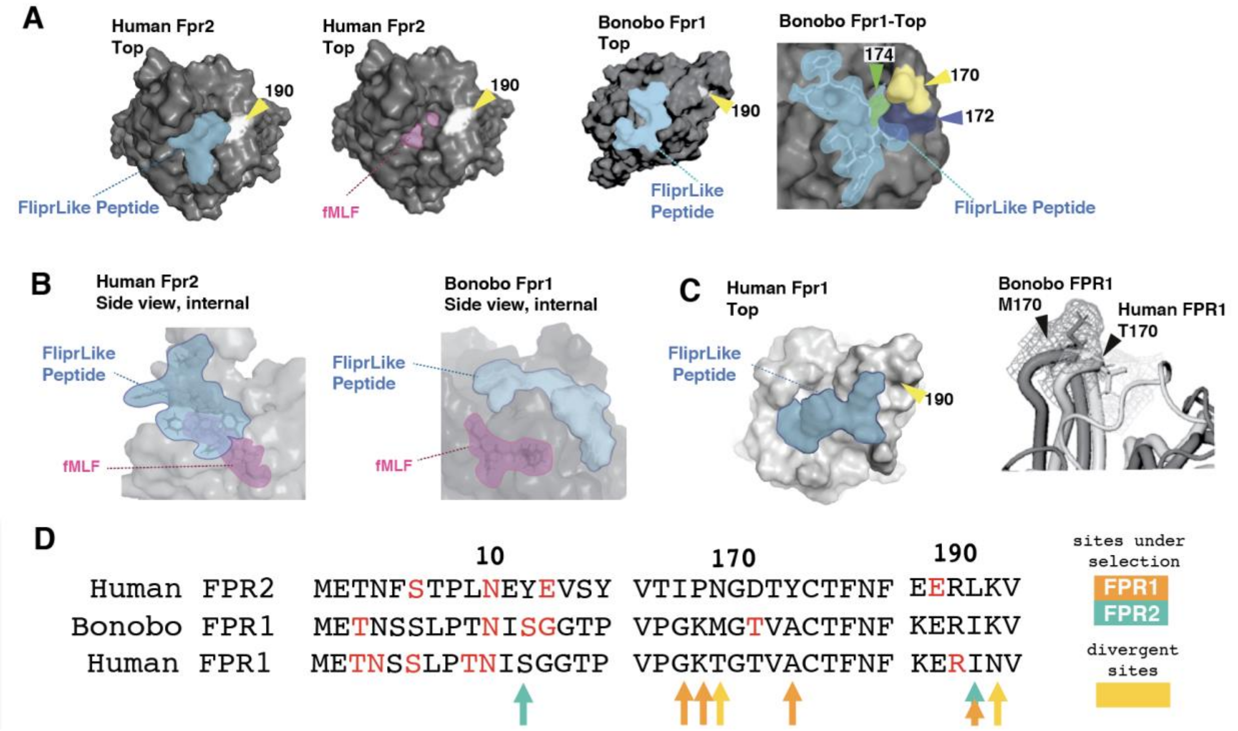

Supplemental Figure 3. Predicted binding interactions between FPRs and bacterial ligands
(A) Molecular modeling of FPR ortholog interactions with formylated peptide (fMLF, pink) or the FLIPr-like peptide (blue). Structures were generated using AlphaFold and docking was performed using Schrodinger Glide. Positions of variable sites in FPRs are indicated. (**B)** Predicted interactions between human FPR2 and bonobo FPR1 with fMLF and FLIPr-like peptide ligands. **(C)** Top view of the human FPR1-FLIPr-like complex. Relative predicted positions of amino acid 170 in bonobo FPR1 and human FPR1 are indicated (right). **(D)** Alignment of variable regions of human FPR1, FPR2, and bonobo FPR1.

FPR1-T170M

Population frequencies of FPR1: T170M	
African/African American	
Ashkenazi Jewish	
European (non-Finnish)	
European (Finnish)	0.0001395
South Asian	0.00009799
East Asian	
Latino	0.0001693
Other	
XX	0.00008499
XY	0.0001044
Total	0.00009550

Data from gnoMAD v2.1.1
Karczewski et al, 2020.
ClinVar (402876)
dbSNP (rs1042229)

FPR1-R190W

Population frequencies of FPR1: R190W	
African/African American	0.08681
Ashkenazi Jewish	0.1176
European (non-Finnish)	0.1284
European (Finnish)	0.162
South Asian	0.186
East Asian	0.1976
Latino	0.08526
Other	0.1292
XX	0.1294
XY	0.1362
Total	0.1330

Data from gnoMAD v2.1.1
Karczewski et al, 2020.
ClinVar (834692)
dbSNP (rs5030880))

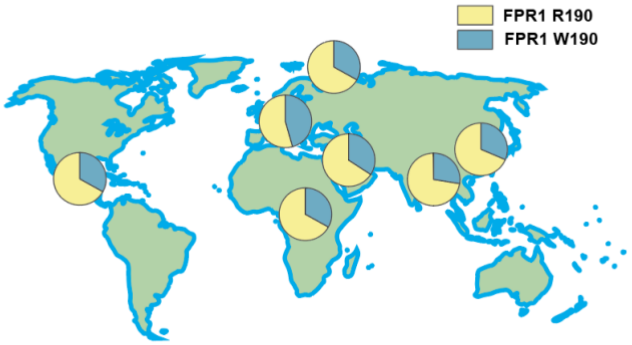

Supplemental Figure 4. Allele frequencies of human *FPR1* polymorphisms. Data obtained from the gnoMAD server.
